# A targeted systematic review of cost analyses for implementation of simulation-based education in healthcare

**DOI:** 10.1177/2050312120913451

**Published:** 2020-03-19

**Authors:** Daniel S Hippe, Rachel A Umoren, Alex McGee, Sherri L Bucher, Brian W Bresnahan

**Affiliations:** 1 University of Washington, Seattle, WA, USA; 2 Indiana University School of Medicine, Indianapolis, IN, USA

**Keywords:** Obstetrics/gynecology, review, simulation, training, cost analysis, cost-effectiveness, neonatal resuscitation

## Abstract

Over the past two decades, there has been an increase in the use of simulation-based education for training healthcare providers in technical and non-technical skills. Simulation education and research programs have mostly focused on the impact on clinical knowledge and improvement of technical skills rather than on cost. To study and characterize existing evidence to inform multi-stakeholder investment decisions, we performed a systematic review of the literature on costs in simulation-based education in medicine in general and in neonatal resuscitation as a particular focus. We conducted a systematic literature search of the PubMed database using two targeted queries. The first searched for cost analyses of healthcare simulation-based education more broadly, and the second was more narrowly focused on cost analyses of neonatal resuscitation training. The more general query identified 47 qualified articles. The most common specialties for education interventions were surgery (51%); obstetrics, gynecology, or pediatrics (11%); medicine, nursing, or medical school (11%); and urology (9%), accounting for over 80% of articles. The neonatal resuscitation query identified five qualified articles. The two queries identified seven large-scale training implementation studies, one in the United States and six in low-income countries. There were two articles each from Tanzania and India and one article each from Zambia and Ghana. Methods, definitions, and reported estimates varied across articles, implying interpretation, comparison, and generalization of program effects are challenging. More work is needed to understand the costs, processes, and outcomes likely to make simulation-based education programs cost-effective and scalable. To optimize return on investments in training, assessing resource requirements, associated costs, and subsequent outcomes can inform stakeholders about the potential sustainability of SBE programs. Healthcare stakeholders and decision makers will benefit from more transparent, consistent, rigorous, and explicit assessments of simulation-based education program development and implementation costs in low- and high-income countries.

## Introduction

In recent decades, there has been a steady increase in the use of simulation-based education (SBE) for training healthcare providers in technical and non-technical skills.^1^ However, much of the focus of simulation education and research programs has been on clinical knowledge, technical skills, and patient outcomes,^2–4^ rather than on economic impact, value, or cost–benefit relationships. Currently, in both high- and low-income countries, institutional training programs, including simulation laboratories, are being scrutinized more carefully for return on investment or costs versus benefits. Policy makers and other stakeholders desire more certainty about expected improvements in provider performance and clinical outcomes linked to SBE. In the coming years, particularly within the context of increased global calls for widespread implementation of universal health coverage,^5^ SBE is likely to become a target of health administrators who will require better evidence of effect and therefore subject these programs to economic evaluation. Educators in the health professions should increase their awareness of potential similarities to bio-pharmaceutical and clinical diagnostic test technology assessments and apply more rigorous assessments of effectiveness as use and costs of SBE increase over time.^6,7^

In anticipation of these expectations for improved efficiency, health outcomes, and return on investment, SBE programs must demonstrate high-quality evidence of their effectiveness and value. Among the SBE approaches to training health workers which include task trainers, manikin-based simulation, and online simulation, virtual reality (VR) simulation platforms are increasing in availability^8,9^ and are an innovative contributor to improving the efficiency of healthcare education, particularly in regard to laparoscopic procedures. Healthcare administrators must make cost-sensitive decisions, first regarding when and how to utilize SBE, and then they must choose between the different approaches to SBE. This is especially true in low- and middle-income countries, where resource constraints are greatest.

In order to study and characterize existing evidence regarding cost implications, cost-effectiveness, and cost–benefit assessment of SBE, we performed a systematic review of the literature on costs in SBE in medicine in general and in neonatal resuscitation as a particular focus. Describing the value of training models and cost implications for training program implementation will inform multi-stakeholder investment decisions.

## Methods

We conducted a literature search for peer-reviewed articles on cost analyses of simulation in healthcare using the MEDLINE® PubMed® databases as of 21 March 2018. Two targeted queries were developed. The general query searched more broadly for cost analyses of healthcare simulation-based training in general. Table 1 shows both queries in full, including the terms used and the logical conjunctives and disjunctives. The general query used terms including “training,” “education,” “high-fidelity simulation,” “virtual reality,” “low dose, high frequency,” “cost analysis,” “cost-effectiveness,” and other similar terms. The focused query included similar terms related to training and cost but also included more specific terms such as “Helping Babies Breathe” (HBB), “Essential Newborn Care” (ENC), “Helping Babies Survive,” “neonatal, and “perinatal” to focus on neonatal resuscitation. The focused query did not simply target a subset of the general query because additional general, less specific terms related to training and cost were included in the focused query as the restriction to neonatal resuscitation substantially limited search results.

**Table 1.** List of sets of terms used in the database search queries.

General query on cost analyses of medical simulation-based training
(((“virtual reality” OR “low dose, high frequency” OR “low dose high frequency”) AND (training OR education OR simulation OR simulator)) OR “simulation training” OR “simulation-based training” OR “simulation-based education” OR ((“high-fidelity simulation” OR “high-fidelity simulator”) AND (training or education))) AND (“cost analysis” OR “cost-effectiveness” OR “economic analysis” OR “economics analysis” OR costing OR “cost benefit analysis”)
Focused query on cost analyses of neonatal resuscitation training
(“Helping babies breathe” OR “essential newborn care” OR “helping babies survive” OR ((“neonatal” OR “perinatal” OR “newborn” OR midwife OR obstetric OR obstetrics) AND resuscitation AND (training OR education OR simulation OR simulator))) AND (“cost analysis” OR “cost-effectiveness” OR “economic analysis” OR “economics analysis” OR costing OR “cost benefit analysis”)

One reviewer reviewed the abstracts of all articles and selected potentially relevant articles for full-text review. To be eligible for review, articles needed to include a cost analysis of a healthcare simulation-based training or education program or tool. The cost analysis could be the focus of the article or only a small part of a larger study. Articles were summarized by article type (original research, research protocol, technical note, systematic review and/or meta-analysis, review/editorial), medical specialty, country of study, and whether VR was used as a training modality. Studies of large-scale implementations of training programs were selected for more detailed review and were each assessed by two reviewers. Additional characteristics of these studies were extracted, including the training program, the scale of the implementation, the cost analysis methodology, cost components covered by the analysis (development, training, equipment, facilities, administration, maintenance), and the conclusion. Except for the cost components, which were coded as present/absent, the other elements extracted were descriptive text due to substantial variation in the types of studies included. The methodology of these studies and potential risk of bias were discussed at the study level.

## Results

**Figure 1.**
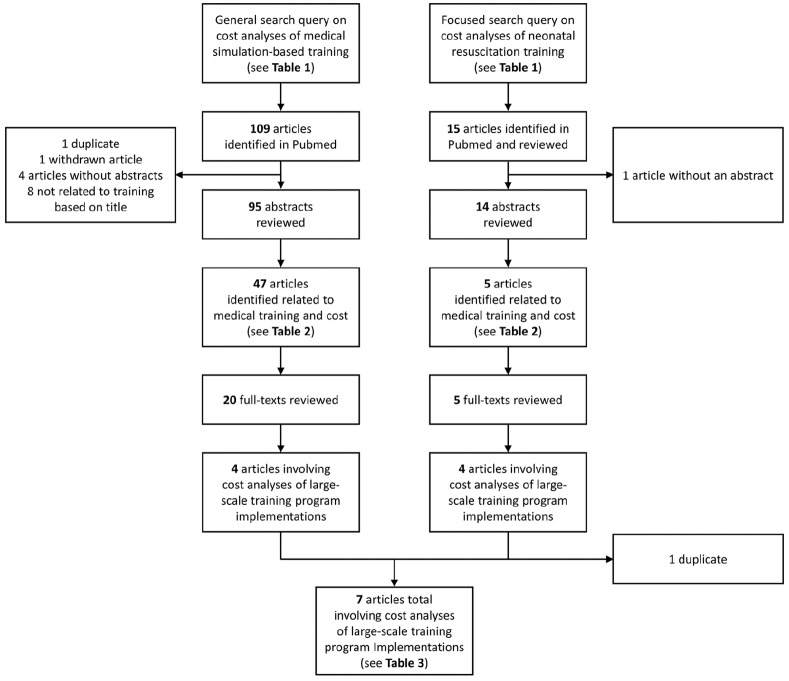
Flowchart of the general (left column) and focused search queries (right column).

The general query for cost analyses of simulation-based training programs identified 109 published articles, while the focused search for cost analyses in the neonatal resuscitation setting identified 16 articles (Figure 1). Of the 109 articles identified in the general search, 47 addressed both cost and medical training. Review of these 47 classified 75% as original research. The most common specialties for education interventions were surgery (51%); obstetrics, gynecology, or pediatrics (11%); general, nursing, or medical school (11%); and urology (9%), accounting for over 80% of articles. Nearly half (49%) of the studies were from the United States, 15% from continental Europe, 11% from Canada, and 9% from the United Kingdom (Table 2). Five (11%) were from low- and middle-income countries. Of the 15 articles identified in the focused search, 5 addressed both cost and medical training. Four (80%) were classified as original research and the other two were classified as research protocols (Table 2). All of these studies were conducted in low- and middle-income countries, including two in Tanzania and one each in India, Ghana, and Zambia.

**Table 2.** Summary of articles from focused and general queries related to medical training and cost.

Variable	Query^a^	
	General (N = 47)	Focused (N = 5)
Article type		
Original Research	35 (74%)	4 (80%)
Research protocol	1 (2%)	1 (20%)
Technical note	1 (2%)	–
Systematic review and/or meta-analysis	1 (2%)	–
Review/editorial	9 (19%)	–
Virtual reality–based training	8 (17%)	–
Department		
Surgery	24 (51%)	–
Obstetrics, gynecology, or pediatrics	5 (11%)	5 (100%)
General, nursing, or medical school	5 (11%)	–
Urology	4 (9%)	–
Anesthesia	3 (6%)	–
Emergency medicine or critical care	3 (6%)	–
Other^b^	3 (6%)	–
Country		
United States	23 (49%)	–
Continental Europe	7 (15%)	–
Canada	5 (11%)	–
United Kingdom	4 (9%)	–
Australia/New Zealand	3 (6%)	–
Tanzania	1 (2%)	2 (40%)
India	1 (2%)	1 (20%)
Ghana	1 (2%)	1 (20%)
Zambia	–	1 (20%)
Egypt	1 (2%)	–
Brazil	1 (2%)	–

– indicates no articles were in the corresponding category.

a One article (Vossius et al.^18^) was identified in both queries.

b Includes diagnostic radiology, interventional radiology, and dentistry.

Across the two queries, there were eight articles on VR-based training that included a cost analysis. These articles predominantly involved surgical subspecialties (n = 6) with a wide variety of training areas. Of the surgical articles, there were three randomized trials for VR training in laparoscopic skills,^10^ endoscopy skills,^11^ and peripheral catheterization skills,^12^ respectively. Another summarized the costs associated with implementing and maintaining an annual 4-week simulation rotation for training surgical skills as part of a general surgery program, although VR was one of the many training modalities utilized.^13^ The remaining two surgical articles were reviews of SBE for urology procedures^14^ and VR simulation in surgical oncology.^15^ Of the two non-surgical articles, one article conducted an economic analysis of a Procedicus Vascular Interventional System Trainer to an animal laboratory for endovascular skills training^16^ and the other was a review of VR simulators in dental education.^17^

As illustrated in Figure 1, a total of seven distinct articles were identified from both queries which reported cost analyses of large-scale training program implementations.^18,13,19–23^ See Table 3 for a detailed summary. Of these articles, one involved a US institution, and each of the six other articles involved low- or middle-income countries. There were two articles from Tanzania, two from India, one from Zambia, and one from Ghana. Several categories or types of cost were described and estimated in these studies. Willcox et al.,^22^ for example, assessed cost and cost-effectiveness of low-dose, high-frequency training in basic emergency obstetric and newborn care in Ghana as compared to standard-of-care approaches in a non-randomized study. They categorized and assessed development costs, start-up costs, and implementation costs. Their cost estimates (2015 US dollar) of four waves of training totaled approximately US$800,000, at roughly US$170,000–US$270,000 for each training wave, with personnel costs consistently being the highest cost category.

**Table 3.** Cost analyses of large-scale training program implementations found in one or both searches.

No.	Article	Country	Training program	Scale	Cost analysis methodology	Cost components included in cost analysis^a^						Total cost (average cost)^b^	Authors’ conclusions
						Development	Training	Equipment	Facilities	Administration	Maintenance		
1	Manasyan *Pediatrics* 2011	Zambia	Essential newborn care training	5 day training 18 midwives trained as trainers, who each trained at their first-level clinics	Actual additional expenses recorded, excluding costs from existing infrastructure		X	X			X	US$20,224 (US$0.98 per delivery)	Low-cost intervention that can reduce early neonatal morality
2	Danzer *J Surg Educ* 2011	United States	Multimodality surgical skills curriculum	Annual 4-week simulation rotation within a general surgery residency program	Costs associated with the curriculum were recorded and categorized		X	X	X		X	US$476,000 (US$12,516 per resident)	The expenditures associated with the program are significant, and this experience may help others develop more cost-effective implementations
3	Vossius *PLoS ONE* 2014	Tanzania	“HBB” training	Initial training and refresher training at one 420-bed hospital	Costs collected from external sources Limited cost perspective, excluding costs borne by other parties		X	X		X	X	US$4431	Low-cost intervention that is cost-effective at a rural hospital
4	Jayanna *PLoS ONE* 2016	India	Onsite mentoring visits, case sheets, and refresher training for managing institutional births and associated complications	Randomized trial of training program involving 108 primary health centers in two districts of Karnataka state	Actual expenditures for implementing the program recorded, categorized into one-time or recurring costs		X	X		X		US$467,371 (US$58,413 per district; US$5.60 per delivery)	Intervention improved facility-readiness and knowledge about diagnosis and management of complications
5	Chaudhury *BMC Health Serv Res* 2016	Tanzania	“HBB” training	2-month implementation + follow-up Mbeya region (49 training sessions, 1341 trainees, 336 health facilities)	Activity-based costing from real-time cost data collection		X	X	X	X	X	US$202,240 (US$4128 per training session; US$151 per trainee; US$602 per health facility)	HBB implementation is a relatively low-cost intervention and nationwide expansion is feasible with substantial investment (>US$3,000,000)
6	Willcox *Global Health* 2017	Ghana	Basic emergency obstetric and newborn care	Two 4-day low-dose training 40 health facilities	Activity-based costing over 3-year time horizon Costs estimated from financial records, interviews, and market prices	X	X	X	X	X	X	US$823,134 (US$20,578 per facility)	The LDHF training approach should be considered for lower cost and efficiency at scale
7	Jeet *Indian J Public Health* 2017	India	General medical skills simulation laboratory	20 permanent and 10 mobile skills laboratory.	Bottom-up costing with health system perspective Financial costs (outlays by program) collected from accounting system Economic costs (incorporating opportunity costs) estimated using interviews to measure activity	X	X	X	X	X	X	US$178,786 (US$139–US$151 per trainee)^c^	Economic implications of skills laboratories should be considered when scaling up in India

HBB: Helping Babies Breath; LDHF: low dose, high frequency.

All costs are expressed in US dollar unless otherwise specified.

a Cost components included are marked with X, while components not included are left blank.

b Total reported cost of implementation; average costs were calculated as the total cost divided by the number of units, where the units available depended on the study and outcomes assessed.

c Costs were reported in Indian Rupees in the original study and were converted to US dollar in this table using a 2012 exchange rate of 1 US dollar = 49.50 Indian Rupees; the original total cost was reported as INR 8,849,895 with the average costs being INR 6856–7474 per trainee.

Manasyan et al.^21^ studied the cost-effectiveness of the World Health Organization’s ENC training in first-level urban settings in Zambia. They assessed two training periods, before and after an ENC program was initiated in Zambian cities. The non-randomized study estimated costs of the training program, approximately US$20,000 (2005 US dollar) to train 18 health workers, roughly US$1100 per worker. They linked program costs to neonatal mortality before and after the ENC training in Zambia (estimated mortality risk reduction of 0.59, or that 97 lives were saved) to estimate cost-effectiveness. Other synchronous educational, quality improvement, and health system changes or initiatives were not adjusted for or included in their study.

Vossius et al.^18^ assessed the cost-effectiveness of a training program, HBB, implemented in Tanzania in a non-randomized setting. Their model included first using the “train-the trainer model” and then HBB refresher training, low-dose high-frequency training, travel, and materials. They estimated an overall training cost of approximately US$4400 for a 2-day training course. Cost estimates did not include training the Master Trainers or expenses for follow-up/refresher training. Vossius and colleagues estimated the number of lives saved linked to HBB training in two distinct 1-year cohorts of patients in Tanzania during 2010–2012, before and after the HBB program was implemented. They extrapolated cost and lives saved effect estimates using a life expectancy approach based on external sources. The Vossius et al. study was not randomized and used a pre- and post-intervention control in a cohort setting, making interpretation of the effect challenging. Chaudhury et al.^19^ focused on estimating costs associated with a large-scale implementation of the HBB program in Tanzania. They included program-specific costs, personnel costs, and capital costs. They estimated activity-based costs for different phases, including initial training and equipment, follow-up training, and central program administration.

Jeet et al.^20^ evaluated simulation-based training of health workers in India and reported the average costs for sequential (repeated) training programs. They estimated the costs of implementing a “skills laboratory” from a health systems perspective. Although their described methods and the link to reported results were not fully transparent, they distinguished between capital costs and recurrent costs in developing estimates of average costs per participant and how average costs changed with additional training events. Their assessment included fixed costs associated with “start-up training” as well as fixed costs of capital investments (buildings, equipment, and repairs). Recurrent costs included personnel, training, monitoring, transportation, consumables, and overhead. They estimated that the average financial costs per participant were 20,000 Indian Rupees (or approximately US$400 per person based on 2012 exchange rate: 1 US dollar = 49.50 Indian Rupees). Regardless of their costing approach used, they estimated that the average costs per participant decrease as more participants are trained. However, they did not address capacity limits or the level of training that is associated with an increase in average costs.

Jayanna et al.^23^ evaluated the effectiveness of a program for improving the quality of institution births at 108 primary health centers in two districts of Karnataka state, India. The study used a parallel, two-arm, cluster randomized design. The intervention consisted of periodic supportive onsite visits by nurse mentors in addition to initial refresher training and new specialized case sheets (documentation of standard procedures and checklists) for maternal and newborn care, which was compared to a similar program without the onsite visits by nurse mentors (control arm). There was significantly greater improvement in facility-readiness for a range of maternal and newborn complications as well as greater improvement in staff knowledge about diagnosis and management of complications in the interventional arm than the control arm. The authors also conducted a cost analysis of the intervention, based on expenditures for rolling out the program in eight districts in the year after the randomized trial was conducted. They estimated the total costs for implementing the program to be US$467,371 in 2013–2014. Start-up (one-time) costs accounted for 12% of the total, with the remaining 88% (US$413,542) being attributed to annual recurring costs including staff salaries and travel, communication and printing, events, and meetings. The estimated cost of implementing the training program was approximately US$5.60 per delivery for 1 year.

The US-based article described the implementation and maintenance of an American College of Surgery/Association of Program Directors in Surgery–based surgical skills curriculum, designed as four 1-week rotations per year within a general surgery residency program.^13^ This article was briefly described earlier due to its utilization of VR simulators as one of several training modalities used, including interactive didactics, case-based discussions, evidence-based handouts, as well as procedural hands-on skills and video training. They included costs associated with space renovations, dedicated personnel and faculty teaching effort, and simulator devices—including breakdowns into initial, maintenance, associated equipment, and replacement costs. Costs associated with designing the curriculum were not reported, and it was unclear whether these expenses were included in the personnel and faculty costs. Administrative costs were not included. The total implementation cost was US$4.2 million between 2006 and 2007. Annual operational expenses were approximately US$476,000 in total, or US$12,516 per medical resident, not including the cost of faculty teaching effort, estimated to be US$30,000 per faculty member. While their study was designed to only analyze cost and not the benefit or impact of their new curriculum, the authors concluded that the associated costs were significant and that more cost-effective approaches may be sought.

## Discussion

Despite the potential benefits associated with SBE programs, cost analyses of their implementation are uncommon in the literature. We conducted a targeted systematic literature review of published evidence of SBE in healthcare settings and specifically SBE in neonatal resuscitation. We identified only seven articles on large-scale implementations from our literature search that we determined to be most relevant. Five of these seven articles were related to maternal and newborn care. In the more general search on SBE, articles reporting cost information typically described small-scale implementations and did not assess long-term administrative and recurring costs associated with implementing or maintaining training programs. Most of the literature does not include randomized controlled designs, a recognized “gold standard” in health-related research.

When costs were reported in SBE studies, there was a lack of consistency and transparency of included components of training development or implementation, making interpretation and comparisons challenging for stakeholders. While we have summarized cost estimates from seven large-scale SBE program implementations in Table 3, it is important to note that these estimates are not necessarily directly comparable to each other and may not generalize to other settings. Several important factors contributing to this are differences in categories of costs assessed; assumptions on training, implementation, maintenance, and recurring costs; the countries where the programs were implemented and their available infrastructure, health systems, and funding systems; the scale of implementation and evaluation, which ranged from a single facility to hundreds; the types of training being delivered; the number of training sessions; and the duration of training. Stakeholders will need to consider these and other factors when interpreting relevant SBE studies and planning future implementations. Furthermore, academic centers in many high-income countries, such as the United States, maintain existing simulation centers where different medical departments and professional groups can receive training on specific skills or tasks. Long-term programmatic or maintenance costs are often subsidized by these medical centers, making it difficult to estimate costs for multi-use SBE centers. The literature in high-income settings has focused more on short-term programmatic costs of conducting a training event or a series of training events. As expected, the bulk of simulation studies in the United States relate to training healthcare providers to perform high-risk and high-cost procedures, such as innovative and/or complex surgical approaches. There are health system and reimbursement incentives to avoid mistakes and reduce complications associated with more expensive or high-risk procedures. These procedures are compelling candidates for additional training or SBE.

Training using simulation in low-income countries continues to evolve and often applies lessons from SBE in high-income countries. At present, SBE in low-income settings tends to focus on basic maternal health, newborn health, and neonatal care processes, such as HBB or ENC and their implementation.^18,19^ However, our systematic evaluation of the SBE cost-related literature indicates that high-quality, long-term studies of training and subsequent care-quality outcomes for trainees or patient health outcomes linked to training are sparse. An additional challenge of cost studies in healthcare is the subsidization of training and clinical care, particularly in low-income countries. In addition, dissemination and implementation of health interventions are often supported by large stakeholders which may be government sponsored, such as the United States Agency for International Development (USAID) or non-profit foundations.

More research is needed in both high- and low-income settings assessing resource requirements, cost categories, cost estimates, benefit estimates, and ranges of costs and benefits related to SBE programs. Zendejas and colleagues^24^ conducted a more broadly defined systematic review focusing on cost studies of simulation training to address the “missing outcome,” that is, costs. They identified cost categories including equipment and materials, personnel, facilities, and other inputs, indicating the number of studies that included each category of cost for simulation training. Equipment, training materials, staff time and cost, and facility rental fees were the most frequently recurring types or categories of costs in the literature.

Several limitations in the existing literature indicate opportunities to improve future research studies in the area of cost analysis for SBE. Researchers can be more specific about assumptions used, perspectives of analyses, sensitivity analyses tested, and potential implications of the assumptions or data limitations. As an example, when cost per lives saved or cost per disability-adjusted life year (averted) outcomes are calculated, as in the study by Manasyan et al.,^21^ the estimates may be based on assumptions of lifetime mortality and disability effects being linked to training a relatively small cohort of instructors to provide subsequent training in their respective public-sector delivery clinics (i.e. train-the-trainer model). Using a reduction in mortality subsequent to a training intervention in a non-randomized study to base estimates of cost per outcome improvement requires caution in interpretation. The mortality reduction may not be directly “caused” by training alone, and it may not be sustainable at a large-scale level. The only randomized trial of a large-scale intervention found in our search was by Jayanna and colleagues,^23^ who concluded that their intervention improved facility readiness and staff knowledge for diagnosing and managing maternal and newborn complications and that their intervention program cost US$413,542 annually to implement in eight high-priority districts in Karnataka, India. However, their methods and results for cost estimates were not clearly described, including multi-year assessment calculations or how the training costs compared to reimbursed amounts or revenue obtained for deliveries at health facilities.

Researchers using standard methods for cost or cost-effectiveness assessment should understand and identify methodological and interpretation-related limitations inherent in these methods. This applies to the entire field of cost-related research and cost-effectiveness assessments, where generalizations are often made without qualifying statements. One major challenge in interpreting economic or cost-related research is that alternative methods are often used. The terminology used may be imprecise or the approaches applied may not be transparent or rigorous. Thus, authors’ interpretations may be over-stated or may not fully recognize uncertainty associated with their study designs and findings.^25^ We recommend that authors clearly state limitations of specific methods, as this will inform stakeholders’ interpretation related to potential generalizability or rationale for increasing the scale or scope of SBE programs. A greater awareness of practical or methodological limitations among journal reviewers and editors will also help to improve the quality of the evidence in the published domain. In some cases, the methods used may not be clearly reported, or potential biases and confounding factors may impact findings and limit generalizability.

Observational study designs may limit interpretations of effects on outcomes or stated limitations may be too narrowly described. Resource use and cost-related analyses, cost-consequence studies, or cost-effectiveness assessments can be conducted in randomized and non-randomized settings and can provide useful information to decision makers when reported with appropriate caveats. Replicating results for specific stakeholder perspectives in multiple studies and the use of sensitivity analyses^26^ will help health education researchers to develop stronger evidence on the costs of SBE with tighter confidence intervals for estimates of ranges of costs compared to benefits. Expanding the collection of “real-world evidence” through implementation research and disseminating the training interventions more broadly will inform the evidence base supporting our global “learning healthcare system.”

Our study had some limitations. We limited our search to MEDLINE and PubMed and did not include non-peer-reviewed literature. This was intended to enable us to identify peer-reviewed articles on the cost of SBE in healthcare in general and in neonatal resuscitation in particular. However, we may have excluded cost data for these interventions reported in articles released by non-profit organizations or government agencies. Our search revealed two research protocols for future randomized controlled trials.^27,28^ It is possible that with the conduct and publication of these studies, there will be additional information available on the costs of SBE. As we sought to describe the publicly available peer-reviewed literature on cost in SBE, we did not contact authors directly for additional data.

## Conclusion

There is increasing pressure to demonstrate the value of SBE (costs vs benefits, or return on investment) to institutions, patients, providers, governments, payers, non-governmental organizations, research sponsors, and other healthcare stakeholders. This includes more conventional SBE and newer innovative simulation-based research and education approaches using virtual and augmented reality simulations. Various stakeholders and decision makers would likely benefit from additional cost assessments of program development, maintenance, and expansion activities associated with transitioning from small-scale to large-scale regional or national implementations across multiple centers.

More work is needed to understand the processes and potential outcomes that make SBE training programs more cost-effective and scalable within and among countries. Stakeholders want to understand resource use implications and options for strengthening the sustainability of SBE. To optimize investments in training, we want to develop high-quality evidence and determine how to enhance the sustainability of SBE models as compelling candidates for providing health worker education opportunities in low-income and high-income settings. To facilitate this, healthcare stakeholders and decision makers would benefit from more rigorous and targeted assessments of resource needs for SBE program development and expansion in multiple settings and from multiple perspectives.
